# *Carica papaya* leaf and root extracts attenuate hyperglycemia-induced insulin resistance by modulating MAPK and PI3K/AKT signalling in hepatic and skeletal muscle cells

**DOI:** 10.1016/j.bbrep.2026.102497

**Published:** 2026-02-13

**Authors:** Mthokozisi Bongani Nxumalo, Rene Bernadette Khan, Nosipho Ntanzi, Fave Yohanna Tata, Hezekiel Mathambo Kumalo

**Affiliations:** Discipline of Medical Biochemistry, School of Laboratory Medicine and Medical Science, University of KwaZulu-Natal, Durban, 4000, South Africa

**Keywords:** *Carica papaya*, Hyperglycemia, MAPK signalling, PI3K/AKT pathway, Insulin resistance, Glucose uptake

## Abstract

Type 2 diabetes mellitus (T2DM) is characterised by impaired glucose homeostasis arising from insulin resistance and inadequate insulin action in peripheral tissues. *Carica papaya* has been reported to exert antidiabetic effects; however, its molecular mechanisms in hepatic and skeletal muscle cells under hyperglycemic conditions remain incompletely understood. This study investigated the effects of *C. papaya* leaf and root extracts on glucose uptake and insulin-related signalling pathways in HepG2 hepatocytes and C2C12 myotubes. Enzyme inhibition assays were used to assess α-amylase and α-glucosidase activity, while protein and gene expression of key components of the MAPK and PI3K/AKT pathways were evaluated using Western blotting and qPCR. *C. papaya* extracts significantly inhibited α-amylase activity (*p* < 0.05), with a non-significant inhibitory trend observed for α-glucosidase, suggesting reduced glucose availability under hyperglycemic conditions. In both HepG2 and C2C12 cells, *C. papaya* attenuated MAPK signalling through suppression of Erk1/2 and p38 MAPK, while JNK inhibition was observed exclusively in HepG2 cells (*p* < 0.05). In HepG2 cells, AKT and GLUT2 gene expression remained unchanged; however, AMPKα and IRS-1 were significantly upregulated, indicating enhanced glucose uptake potential despite a concomitant reduction in glycogen synthase expression (*p* < 0.05). In contrast, C2C12 myotubes exhibited enhanced insulin signalling characterised by increased phosphorylated IRS-1, AKT activation, and elevated glycogen synthase expression, supporting improved glucose uptake and storage (*p* < 0.05). Collectively, these findings demonstrate that *C. papaya* extracts mitigate hyperglycemia-induced insulin resistance by suppressing MAPK signalling and enhancing glucose uptake through distinct, cell-specific mechanisms in hepatic and skeletal muscle cells.

## Highlights

• *C. papaya* extracts.
• Alleviate insulin resistance.
• Enhanced glucose uptake.

## 1 Introduction

Diabetes mellitus (DM) is a chronic metabolic disorder characterised by persistent hyperglycemia arising from impaired insulin secretion, insulin action, or both [1]. The global burden of diabetes continues to escalate at an alarming rate. According to the International Diabetes Federation (IDF), approximately 589 million adults aged 20–79 years are projected to be living with diabetes by 2024, contributing to an estimated 3.4 million deaths, which represents 9.3% of all global mortality [2]. By 2050, the number of individuals affected by diabetes is expected to rise to 853 million, with an estimated 252 million adults remaining undiagnosed, highlighting a substantial and growing public health challenge. This increasing prevalence places a significant strain on healthcare systems worldwide, particularly in middle-income countries such as South Africa, where rapid urbanisation and lifestyle transitions exacerbate disease risk [3].

The liver plays a central role in maintaining glucose homeostasis by regulating glucose uptake, storage, and production [4]. Hepatic glucose uptake occurs largely independently of insulin and is primarily mediated by the glucose transporter GLUT2 [5]. Under normoglycemic conditions, glucose entering hepatocytes is stored as glycogen through glycogenesis, a process tightly regulated by glycogen synthase and its upstream inhibitor, glycogen synthase kinase-3 (GSK3) [6,7]. During fasting or energy-deprived states, the liver initiates glycogenolysis and gluconeogenesis to maintain circulating glucose levels [4]. However, under chronic hyperglycemic conditions, excessive glucose influx promotes de novo lipogenesis, leading to hepatic triglyceride accumulation, insulin resistance, and non-alcoholic fatty liver disease [[8], [9], [10]]. Impaired hepatic insulin signalling further results in inappropriate glucose production, contributing significantly to fasting hyperglycemia in type 2 diabetes mellitus (T2DM) [10,11].

Skeletal muscle is the primary site of postprandial glucose disposal, accounting for approximately 70–80% of insulin-stimulated glucose uptake [12]. Unlike the liver, glucose entry into skeletal muscle is highly insulin-dependent and is mediated by the translocation of glucose transporter type 4 (GLUT4) to the cell membrane [13]. This process is regulated predominantly through the phosphoinositide-3-kinase/protein kinase B (PI3K/AKT) signalling pathway, initiated by insulin binding to the insulin receptor and subsequent phosphorylation of insulin receptor substrate-1 (IRS-1) [14]. Activation of AKT promotes GLUT4 translocation and stimulates glycogen synthase, facilitating glucose storage as glycogen [13,15]. Disruption of this pathway impairs glucose uptake and is a hallmark of skeletal muscle insulin resistance [16].

Insulin resistance in both hepatic and skeletal muscle tissues is further exacerbated by chronic activation of stress-responsive signalling pathways, particularly the mitogen-activated protein kinases (MAPKs), including *c*-Jun N-terminal kinase (JNK), extracellular signal-regulated kinase (ERK1/2), and p38 MAPK [12,17]. Sustained MAPK activation promotes inhibitory serine phosphorylation of IRS-1, attenuating insulin signalling and reducing glucose uptake [18,19]. In parallel, the 5′-adenosine monophosphate-activated protein kinase (AMPK), a key cellular energy sensor, becomes dysregulated in T2DM [20]. Under physiological conditions, AMPK activation enhances glucose uptake, suppresses hepatic glucose production, and restores energy balance. However, impaired AMPK signalling contributes to defective glucose utilisation and metabolic inflexibility in insulin-resistant states [12,17].

Current therapeutic strategies for T2DM primarily focus on lifestyle modification combined with pharmacological interventions such as metformin, insulin secretagogues, and insulin therapy [21]. While metformin remains the first-line treatment due to its efficacy and affordability, its long-term use is associated with adverse effects, including gastrointestinal intolerance, vitamin B12 deficiency, and rare cases of lactic acidosis [16,22,23]. Moreover, these therapies primarily manage glycaemia rather than reversing the underlying molecular mechanisms of insulin resistance, underscoring the need for safer and more effective alternatives [16].

*Carica papaya* is a medicinal plant widely used in traditional medicine and is recognised for its antioxidant, anti-inflammatory, and antidiabetic properties [[24], [25], [26], [27]]. Its therapeutic potential is attributed to a rich phytochemical composition, including flavonoids, phenolic compounds, alkaloids, carotenoids, and glucosinolates. Although previous studies have demonstrated the glucose-lowering effects of *C. papaya* in experimental models of diabetes, the molecular mechanisms underlying its actions in key metabolic tissues remain poorly defined [28,29]. Notably, limited information exists regarding its comparative effects on hepatic and skeletal muscle glucose uptake pathways under hyperglycemic conditions.

Therefore, this study aimed to investigate and compare the effects of *C. papaya* leaf and root extracts on glucose-regulatory enzymes and intracellular signalling pathways involved in glucose uptake and insulin sensitivity in HepG2 liver cells and C2C12 skeletal muscle cells under hyperglycemic conditions, using metformin as a reference control.

## 2 Materials and methods

### 2.1 Materials

HepG2 and C2C12 cell lines were obtained from Highveld Biological (Johannesburg, South Africa). Cell culture media, supplements, and consumables were purchased from Whitehead Scientific (Johannesburg, South Africa). α-Amylase and α-glucosidase activity assay kits were obtained from Sigma-Aldrich (Merck, Darmstadt, Germany). Western blot reagents were purchased from Bio-Rad (Hercules, CA, USA), and antibodies were sourced from Cell Signalling Technology (Danvers, MA, USA) via Anatech (Johannesburg, South Africa). All other reagents were of analytical grade.

### 2.2 Cell culture and hyperglycemic model

The HepG2 and C2C12 cells were cultured in Dulbecco's Modified Eagle Medium (DMEM) supplemented with 10% foetal calf serum, 1% penicillin–streptomycin–fungizone, and 1% l-glutamine. Cells were maintained at 37 °C in a humidified incubator with 5% CO_2_ and subcultured at ∼70% confluence. To synchronise metabolic responses, cells were serum-starved for 8 h before treatment. Hyperglycemia was induced by incubating cells in high-glucose (25 mM) DMEM for 18 h, while normoglycemic controls received low-glucose (5 mM) DMEM. This model was selected to mimic chronic hyperglycemic conditions observed in T2DM.

### 2.3 Preparation of *Carica papaya* extracts

Fresh *Carica papaya* leaves and roots were shade-dried and ground into a fine powder. Thereafter, 10 g of leaf powder and root powder were separately extracted in deionised water (150 mL and 200 mL, respectively) under continuous stirring for 2 h. Extracts were centrifuged at 2000×*g* for 10 min, and the supernatants were lyophilised and stored at 4 °C. Working concentrations (500 and 1000 μg/mL) were prepared from a 5 mg/mL stock solution.

### 2.4 Treatment conditions

Cells were treated for 24 h under the following conditions: normoglycemic control, hyperglycemic control, metformin (100 μg/mL), *C. papaya* leaf extract (500 and 1000 μg/mL), and *C. papaya* root extract (500 and 1000 μg/mL). Concentrations were selected based on previous cytotoxicity and efficacy studies [30].

### 2.5 The MTT assay

The MTT (3-(4,5-dimethylthiazol-2-yl)-2,5-diphenyl-2H-tetrazolium bromide) colorimetric assay was utilised to assess the metabolic activity of viable cells in C2C12 skeletal muscle cell lines, as previously done in HepG2 [30]. A suspension of C2C12 cells was seeded into a 96-well microtiter plate at a concentration of 20,000 cells per well (200 μL/well) in triplicate. The cells were allowed to adhere overnight at 37 °C in an atmosphere of 5% CO_2_. After starvation (8 h) and induction (18 h), the cells were treated with concentrations of *C. papaya* extracts (500 and 1000 μg/mL) and with NG, HG and metformin (100 μg/mL). The plate was then incubated for 24 h under the same conditions (37 °C, 5% CO_2_). After incubation, the treatment samples were removed and replaced with 20 μL of a 5 mg/mL MTT salt solution (in 0.1 M PBS) along with 100 μL of CCM, and the cells were incubated again at 37 °C with 5% CO_2_. After 4 h, the MTT salt solution was discarded, and 100 μL of dimethyl sulfoxide (DMSO) was added to each well. The plate was incubated for an additional hour at 37 °C with 5% CO_2_ to solubilise the formazan crystals. Absorbance values were quantified using a SPECTROstar® Nano microplate reader (BMG LABTECH, Ortenberg, Germany) at 570 nm, with a reference wavelength of 690 nm. These absorbance values were used to calculate the percentage of cell viability relative to the control.

### 2.6 The α-amylase assay

α-Amylase activity was measured using a commercially available colorimetric assay kit (MAK478, Sigma-Aldrich, Merck KGaA, Germany) according to the manufacturer's instructions, with minor adaptations for cell culture–derived samples. This assay is based on the hydrolysis of starch by α-amylase, followed by enzymatic conversion of the released glucose to hydrogen peroxide, which is quantified colorimetrically at 585 nm.

To eliminate endogenous glucose, samples were processed using a 10 kDa centrifugal filter unit (Microcon Ultracel-10). Briefly, 50 μL of the sample was loaded into the filter unit, diluted with 500 μL assay buffer, and centrifuged at 14,000×*g* for 30 min at room temperature. The procedure was repeated once, and the final sample volume was recorded to calculate the dilution factor (DF).

Following glucose removal, 10 μL of each prepared sample was added to a clear flat-bottom 96-well plate in triplicate. A 400 mg/dL glucose standard was prepared according to the manufacturer's protocol and included in parallel. Assay buffer served as the blank control. The working reagent was freshly prepared and added to each well, followed by incubation at 25 °C for 15 min. Detection reagent was then added, and the plate was incubated for an additional 20 min at 25 °C before absorbance was measured at 585 nm. α-Amylase activity was calculated using the following equation:

$$\alpha ‐\text{Amylase}\;\left(U/L\right)=\frac{\left({\text{OD}}_{\text{Sample}}-{\text{OD}}_{\text{Blank}}\right)}{\left({\text{OD}}_{\text{Standard}}-{\text{OD}}_{\text{Blank}}\right)}\times \frac{400}{T}\times \text{DF}$$

where *T* represents the incubation time (minutes), and *DF* denotes the sample dilution factor. One unit of α-amylase activity was defined as the amount of enzyme required to generate 1 μmol of glucose per minute under the assay conditions.

### 2.7 α-Glucosidase activity assay

α-Glucosidase activity was measured using a commercial colorimetric assay kit (MAK123, Sigma-Aldrich, Merck KGaA, Germany) according to the manufacturer's instructions. This kinetic assay is based on the hydrolysis of *p*-nitrophenyl-α-d-glucopyranoside (α-NPG) by α-glucosidase, resulting in the formation of *p*-nitrophenol, which is quantified at 405 nm.

Briefly, 20 μL of each sample was added to a clear flat-bottom 96-well plate. Ultrapure water was used as the blank, and the supplied calibrator (equivalent to 250 U/L) was included for activity calculation. A freshly prepared master reaction mix consisting of assay buffer and α-NPG substrate was added to each well, and the plate was gently mixed. Initial absorbance was recorded at 405 nm, followed by incubation at 37 °C for 20 min, after which the final absorbance was measured.

α-Glucosidase activity was calculated based on the change in absorbance over time (ΔA405/min) relative to the calibrator, as per the manufacturer's guidelines. One unit of α-glucosidase activity was defined as the amount of enzyme that catalyses the hydrolysis of 1 μmol of substrate per minute at pH 7.0.

Although the assay is primarily validated for biological samples, it was successfully adapted for use with cell culture–derived samples. Enzyme activity values were normalised and expressed as fold change relative to the hyperglycemic control.

### 2.8 Western blotting

#### 2.8.1 Protein isolation

The HepG2 and C2C12 cells were treated as described and washed three times with ice-cold 0.1 M phosphate-buffered saline (PBS). Cells were lysed by the addition of 600 μL Cytobuster™ reagent (Novagen, San Diego, CA, USA) supplemented with phosphatase and protease inhibitor mixtures (04906837001 and 05892791001, respectively; Roche, Germany). Flasks were incubated on ice for 30 min, after which cells were scraped and lysates transferred to 1.5 mL microcentrifuge tubes. Samples were centrifuged at 10,000×*g* for 10 min at 4 °C, and the resulting supernatants containing total cellular protein were collected for downstream analysis.

#### 2.8.2 Protein quantification and standardisation

Protein concentrations were determined using the bicinchoninic acid (BCA) assay. Bovine serum albumin (BSA) standards were prepared at concentrations of 0, 0.2, 0.4, 0.6, 0.8, and 1 mg/mL. Thereafter, 25 μL of each standard and protein sample were dispensed into a 96-well plate in triplicate. A working BCA reagent was prepared by mixing BCA solution with copper sulfate (CuSO_4_) according to the manufacturer's instructions, and 200 μL was added to each well. Plates were incubated at 37 °C for 30 min, and absorbance was measured at 562 nm using a SPECTROstar Nano microplate reader (BMG Labtech, Ortenberg, Germany). Protein concentrations were calculated from the standard curve, and all samples were standardised to 1 mg/mL. Standardised proteins were mixed with Laemmli sample buffer (containing SDS, β-mercaptoethanol, glycerol, Tris-HCl, and bromophenol blue) at a 1:4 ratio, boiled at 100 °C for 5 min, cooled, and stored at −80 °C until analysis.

#### 2.8.3 SDS-PAGE

Proteins were separated by SDS-PAGE using the MINI-PROTEAN® Tetra Cell system (Bio-Rad). Ten percent resolving gels and 4% stacking gels were prepared using standard protocols. Equal volumes (25 μL) of denatured protein samples were loaded into each well, and electrophoresis was performed at 150 V for 90 min in 1 × running buffer (Tris–glycine, pH 8.3).

#### 2.8.4 Protein transfer and immunoblotting

Following electrophoresis, proteins were transferred onto nitrocellulose membranes using the Trans-Blot Turbo Transfer System (Bio-Rad, CA, USA) for 30 min at 25 V. Membranes were blocked for 2 h at room temperature in 5% bovine serum albumin (BSA) prepared in Tris-buffered saline with 0.05% Tween-20 (TTBS, pH 7.5). Membranes were incubated with primary antibodies (Table 1), diluted 1:1000 in 2% BSA/TTBS, for 1 h at room temperature followed by overnight incubation at 4 °C. Membranes were washed five times with TTBS (10 min per wash) and incubated with horseradish peroxidase (HRP)-conjugated secondary antibody (1:2500 in 2% BSA/TTBS) for 2 h at room temperature. Following five additional washes, immunoreactive proteins were visualised using Clarity™ Western ECL substrate (Bio-Rad) and imaged using the Chemidoc™ Imaging System (Bio-Rad).

**Table 1:** Primary and secondary antibodies used for immunoblotting.

Target protein	Antibody type	Host species	Catalogue number	Supplier	Dilution
SAPK/JNK	Total	Rabbit	9252S	Cell Signaling Technology (USA)	1:1000
Phospho-SAPK/JNK (Thr183/Tyr185)	Phospho-specific	Rabbit	9251S	Cell Signaling Technology (USA)	1:1000
p44/42 MAPK (ERK1/2)	Total	Rabbit	4348S	Cell Signaling Technology (USA)	1:1000
Phospho-p44/42 MAPK (Thr202/Tyr204)	Phospho-specific	Rabbit	8544S	Cell Signaling Technology (USA)	1:1000
p38 MAPK	Total	Rabbit	8690S	Cell Signaling Technology (USA)	1:1000
Phospho-p38 MAPK (Thr180/Tyr182)	Phospho-specific	Rabbit	4511S	Cell Signaling Technology (USA)	1:1000
AMPKα	Total	Rabbit	2532S	Cell Signaling Technology (USA)	1:1000
Phospho-AMPKα (Thr172)	Phospho-specific	Rabbit	2535S	Cell Signaling Technology (USA)	1:1000
IRS-1	Total	Rabbit	2382S	Cell Signaling Technology (USA)	1:1000
Phospho-IRS-1 (Ser101)	Phospho-specific	Rabbit	2385S	Cell Signaling Technology (USA)	1:1000
AKT	Total	Rabbit	9272S	Cell Signaling Technology (USA)	1:1000
Glycogen synthase	Total	Rabbit	3886S	Cell Signaling Technology (USA)	1:1000
β-actin	Loading control	Mouse	A0bD12141	Sigma-Aldrich (Merck)	1:5000
Anti-rabbit IgG–HRP	Secondary	Goat	7074	Cell Signaling Technology (USA)	1:2500

#### 2.8.5 β-Actin probing and densitometric analysis

To probe for the housekeeping protein β-actin, membranes were stripped using 5% hydrogen peroxide at 37 °C for 30 min to quench residual HRP activity, followed by washing with TTBS and re-blocking with 5% BSA for 1 h. Membranes were incubated with anti-β-actin antibody for 1 h at room temperature, washed, and re-imaged using the Chemidoc™ system.

Band intensities were quantified using Image Lab software (Bio-Rad) and normalised to β-actin. Data are presented as mean relative band density (RBD).

All primary antibodies were diluted in 2% bovine serum albumin (BSA) in Tris-buffered saline containing 0.05% Tween 20 (TTBS). Protein expression levels were normalised to β-actin.

### 2.9 Quantitative polymerase chain reaction (qPCR)

#### 2.9.1 RNA isolation

Following treatment, culture media were aspirated, and cells were washed once with phosphate-buffered saline (PBS). Total RNA was extracted using TRIzol™ reagent (Invitrogen) according to the manufacturer's protocol. Briefly, 500 μL of TRIzol was added to each flask, and cells were detached using a sterile cell scraper. Lysates were transferred to RNase/DNase-free microcentrifuge tubes and stored overnight at −80 °C.

Samples were thawed on ice, and 100 μL of chloroform was added, followed by incubation at room temperature for 3 min. Phase separation was achieved by centrifugation at 12,000×*g* for 15 min at 4 °C. The aqueous phase was transferred to a fresh RNase/DNase-free tube, mixed with 250 μL of isopropanol, and incubated overnight at −80 °C. RNA was pelleted by centrifugation at 12, 000×*g* for 20 min at 4 °C, washed with 75% ice-cold ethanol, and centrifuged at 7400×*g* for 15 min. The RNA pellet was air-dried and resuspended in 15 μL of nuclease-free water.

#### 2.9.2 RNA quantification and standardisation

RNA concentration and purity were assessed using a NanoDrop™ 2000 spectrophotometer (Thermo Fisher Scientific, USA). Absorbance ratios (A260/A280) were recorded to confirm RNA integrity. Samples were standardised to a final concentration of 100 ng/μL using nuclease-free water.

#### 2.9.3 cDNA synthesis

Complementary DNA (cDNA) was synthesised using the Maxima H Minus First Strand cDNA Synthesis Kit (Thermo Fisher Scientific) according to the manufacturer's instructions. For each reaction, 1 μg of total RNA was combined with a primer mix containing oligo (dT)18 and random hexamers. Reverse transcription was performed using a GeneAmp PCR System with the following conditions: 25 °C for 10 min, 52 °C for 30 min, and enzyme inactivation at 85 °C for 5 min cDNA samples were diluted with nuclease-free water and stored at −80 °C until analysis.

#### 2.9.4 Quantitative PCR

Quantitative PCR was performed using SYBR Green chemistry on the Applied Biosystems™ QuantStudio™ Real-Time PCR System. Each reaction (10 μL total volume) consisted of SYBR Green master mix (5 μL), forward primer (1 μL), reverse primer (1 μL), nuclease-free water (1.5 μL), and cDNA template (1.5 μL). Reactions were run in triplicate.

Thermal cycling conditions were as follows: initial denaturation at 95 °C for 5 min, followed by 40 cycles of denaturation at 95 °C for 15 s, gene-specific annealing (Table 2), and extension at 72 °C for 2 s. Melt curve analysis was performed to confirm amplification specificity.

**Table 2:** Genes of interest, primer sequences and annealing temperatures.

Gene	Primer sequence (5′–3′)	Annealing temperature (°C)
AKT	F: GCATAAGCCTGAACCAAGC R: GACAGAGACGGAGGGTGACG	59
GAPDH	F: TCCCTGAGCTGAACGGGAAG R: GGAGGAGTGGGTGTCGCTGT	58
GLUT2	F: GGCTAATTTCAGGACTGGTT R: TTTCTTTGCCCTGACTTCCT	57

#### 2.9.5 Data analysis

Gene expression levels were normalised to the housekeeping gene GAPDH, which was amplified under identical conditions. Relative mRNA expression was calculated using the 2^−ΔΔCt^ method (Livak and Schmittgen, 2001), with results expressed as fold change relative to the hyperglycemic control.

### 2.10 Statistical analysis

All experiments were performed in triplicate and repeated at least three times independently. Data are presented as mean ± SEM. Statistical significance was determined using one-way ANOVA followed by appropriate post hoc tests, with *p* < 0.05 considered statistically significant.

## 3 Results

### 3.1 Cell viability (C2C12)

**Fig. 1:**
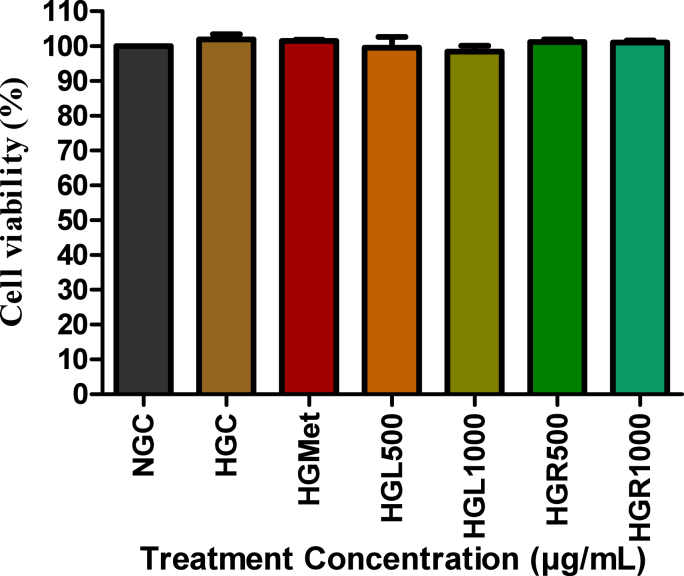
**Cell viability of HepG2 and C2C12 cells following treatment with *Carica papaya* leaf and root extracts under hyperglycemic conditions.** Cell viability was assessed after treatment with *C. papaya* leaf (HGL500, HGL1000), root (HGR500, HGR1000) extracts, metformin, or high-glucose control (HG). Data are mean ± SEM of three independent experiments performed in triplicate. One-way ANOVA with post hoc testing; *p* < 0.05 vs HG.

Treatment of hyperglycemia-induced C2C12 cells with *C. papaya* leaf and root extracts at 500 and 1000 μg/mL did not significantly affect cell viability. Cell viability remained between 98 and 102% relative to normoglycemic controls and was comparable to metformin-treated cells (100 μg/mL; *p* > 0.05) (Fig. 1). No statistically significant differences were observed between treated groups, normoglycemic controls (5 mM glucose), or hyperglycemic controls (25 mM glucose), confirming the non-cytotoxic nature of the extracts under the experimental conditions.

### 3.2 The α-glucosidase and α-amylase

#### 3.2.1 α-Amylase activity

**Fig. 2:**
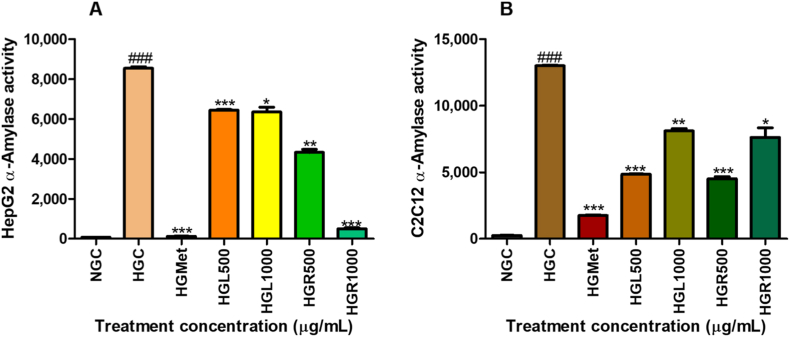
**Effect of *Carica papaya* extracts on α-amylase activity in (A) HepG2 and (B) C2C12 cells.** α-Amylase activity was determined using a colorimetric assay and calculated according to the manufacturer's protocol. Data are expressed as fold change relative to the hyperglycemic control and presented as mean ± SEM (*n* = 3). *p* < 0.05 vs hyperglycemic control.

In HepG2 cells, α-amylase activity was significantly elevated under hyperglycemic conditions (8537 ± 74.25 U/L) compared to normoglycemic controls (78.52 ± 0.00 U/L; *p* < 0.0001) (Fig. 2A). Treatment with *Carica papaya* significantly reduced α-amylase activity at all tested concentrations, including HGL500 (6434 ± 40.38 U/L; 0.75-fold; *p* = 0.0001), HGL1000 (6349 ± 237.9 U/L; 0.74-fold; *p* = 0.0127), HGR500 (4319 ± 153.5 U/L; 0.51-fold; *p* = 0.0016), and HGR1000 (501.2 ± 48.84 U/L; 0.06-fold; *p* < 0.0001), relative to the hyperglycemic control. Metformin treatment produced a comparable reduction in α-amylase activity (118.5 ± 8.553 U/L; *p* < 0.0001).

Similarly, in C2C12 cells, hyperglycaemia significantly increased α-amylase activity (12,990 ± 54.80 U/L) compared to normoglycemic controls (247.6 ± 11.00 U/L; *p* < 0.0001) (Fig. 2B). Metformin treatment reduced α-amylase activity to 1750 ± 44.48 U/L (0.13-fold; *p* < 0.0001). Treatment with *C. papaya* leaf and root extracts also significantly decreased α-amylase activity at HGL500 (4840 ± 32.38 U/L; 0.37-fold; *p* < 0.0001), HGL1000 (8093 ± 176.3 U/L; 0.62-fold; *p* = 0.0014), HGR500 (4505 ± 167.2 U/L; 0.35-fold; *p* = 0.0004), and HGR1000 (7608 ± 718.6 U/L; 0.59-fold; *p* = 0.0175), relative to the hyperglycemic control.

#### 3.2.2 α-Glucosidase activity

**Fig. 3:**
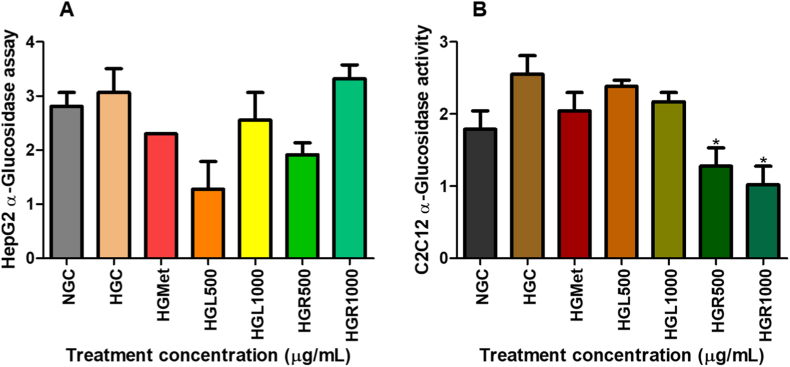
**Effect of *Carica papaya* extracts on α-glucosidase activity in (A) HepG2 and (B) C2C12 cells.** Enzyme activity was assessed using a kinetic colorimetric assay and calculated according to the manufacturer's instructions. Data are expressed as fold change relative to the hyperglycemic control and presented as mean ± SEM (*n* = 3). No statistically significant differences were observed (*p* > 0.05).

The α-glucosidase activity in HepG2 cells exhibited a non-significant increase under hyperglycemic conditions (3.064 ± 0.4423 U/L) compared to normoglycemic controls (2.809 ± 0.2554 U/L; *p* > 0.05) (Fig. 3A). Neither metformin treatment (2.298 ± 0.0000 U/L; *p* = 0.2254) nor *C. papaya* extracts at HGL500 (1.277 ± 0.5107 U/L; *p* = 0.0773), HGL1000 (2.554 ± 0.5107 U/L; *p* = 0.5046), or HGR500 (1.915 ± 0.2212 U/L; *p* = 0.1458) produced statistically significant changes relative to the hyperglycemic control.

In C2C12 cells, α-glucosidase activity was increased under hyperglycemic conditions (2.554 ± 0.2554 U/L) compared to normoglycemic controls (1.788 ± 0.2554 U/L; *p* = 0.1240) (Fig. 3B). While metformin and *C. papaya* leaf extracts did not significantly alter enzyme activity (*p* > 0.05), treatment with *C. papaya* root extract resulted in significant reductions at HGR500 (1.277 ± 0.2554 U/L; 0.50-fold; *p* = 0.0385) and HGR1000 (1.021 ± 0.2554 U/L; 0.40-fold; *p* = 0.0240) relative to the hyperglycemic control.

### 3.3 Modulation of MAPK signalling pathways

#### 3.3.1 The JNK protein expression in HepG2 and C2C12 cells

**Fig. 4:**
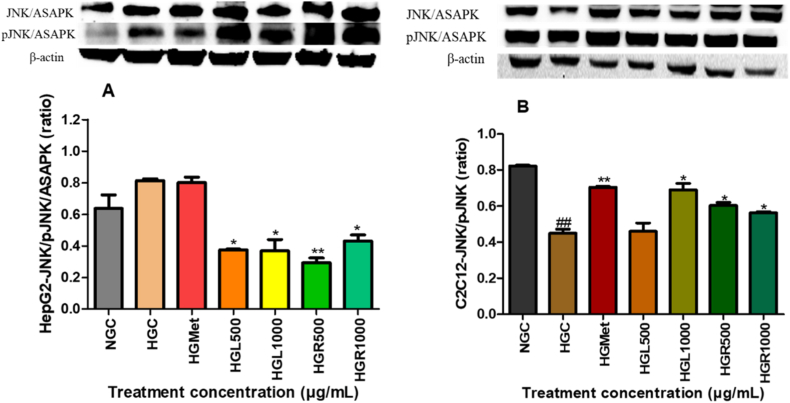
**Regulation of SAPK/JNK and phosphorylated SAPK/JNK by *Carica papaya* extracts in (A) HepG2 and (B) C2C12 cells.** Protein expression was analysed by Western blotting following treatment under high-glucose conditions. Band intensities were normalised to β-actin and expressed as mean relative band density (RBD) ± SEM (n = 3). *p* < 0.05 vs HG.

Hyperglycemic conditions significantly increased SAPK/JNK expression in HepG2 cells (1.45 ± 0.08-fold) compared to normoglycemic controls (1.00 ± 0.05; *p* < 0.01) (Fig. 4A). Treatment with *C. papaya* leaf extract significantly reduced SAPK/JNK expression at HGL500 (0.88 ± 0.04-fold; *p* = 0.0032) and HGL1000 (0.79 ± 0.06-fold; *p* = 0.0011). Similar reductions were observed with root extract at HGR500 (0.91 ± 0.07-fold; *p* = 0.0048) and HGR1000 (0.74 ± 0.05-fold; *p* < 0.0001). Metformin treatment did not significantly alter SAPK/JNK expression relative to the hyperglycemic control (*p* > 0.05).

In C2C12 cells, JNK expression was significantly increased under several treatment conditions relative to hyperglycemic controls (HGC) (Fig. 4B). Metformin treatment resulted in a significant increase in JNK expression (1.57 ± 0.01-fold; *p* = 0.0078). Similarly, *C. papaya* leaf extract at HGL1000 increased JNK expression to 1.53 ± 0.08-fold (*p* = 0.0103), while root extract induced significant increases at HGR500 (1.34 ± 0.04-fold; *p* = 0.0131) and HGR1000 (1.25 ± 0.01-fold; *p* = 0.0396). In contrast, treatment with HGL500 did not significantly alter JNK expression (1.02 ± 0.10-fold; *p* = 0.8686). Notably, hyperglycemia alone significantly reduced JNK expression in HGC cells (0.55 ± 0.03-fold) relative to normoglycemic controls (NGC; *p* = 0.0036).

#### 3.3.2 The Erk (p42/44 MAPK) protein expression in HepG2 and C2C12 cells

**Fig. 5:**
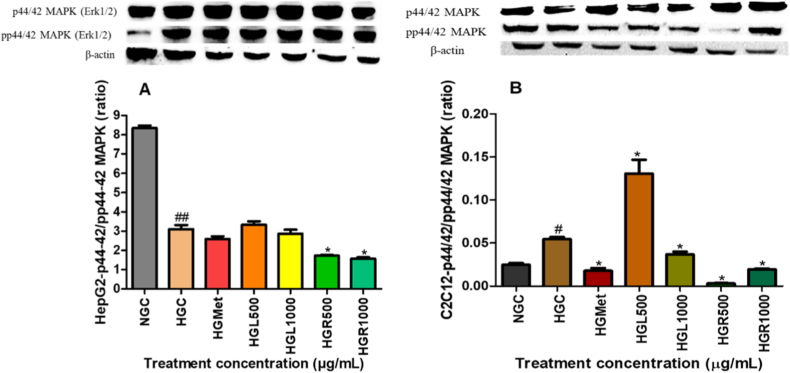
**Effects of *Carica papaya* extracts on p44/42 MAPK (Erk1/2) and phosphorylated Erk1/2 in (A) HepG2 and (B) C2C12 cells.** Western blot analysis was performed after treatment under hyperglycemic conditions. Densitometric values were normalised to β-actin and expressed as mean ± SEM (n = 3). *p* < 0.05 vs HG.

Erk1/2 expression was significantly decreased in hyperglycemic HepG2 cells (1.38 ± 0.07-fold) relative to normoglycemic controls (*p* = 0.0023) (Fig. 5A). Treatment with *C. papaya* root extract significantly reduced Erk1/2 expression at HGR500 (0.89 ± 0.05-fold; *p* = 0.0087) and HGR1000 (0.82 ± 0.06-fold; *p* = 0.0019), whereas leaf extract and metformin did not produce statistically significant changes (*p* > 0.05).

Hyperglycemic conditions significantly increased Erk1/2 expression in C2C12 cells (1.54 ± 0.08-fold; *p* < 0.001) compared to normoglycemic controls (Fig. 5B). Treatment with *C. papaya* leaf extract at HGL1000 significantly reduced Erk1/2 expression to 0.93 ± 0.06-fold (*p* = 0.0043), while root extract induced comparable reductions at HGR500 (0.88 ± 0.05-fold; *p* = 0.0017) and HGR1000 (0.91 ± 0.04-fold; *p* = 0.0039). Metformin similarly reduced Erk1/2 expression (0.91 ± 0.07-fold; *p* = 0.0065). In contrast, treatment with HGL500 resulted in a further elevation of Erk1/2 expression (1.67 ± 0.11-fold; *p* = 0.0214) relative to hyperglycemic controls.

#### 3.3.3 The protein expression of p38 in HepG2 and C2C12 cells

**Fig. 6:**
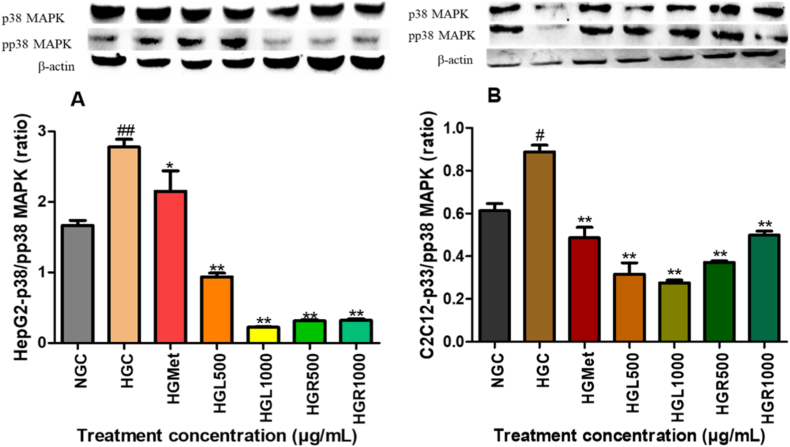
**Modulation of p38 MAPK and phosphorylated p38 MAPK by *Carica papaya* extracts in (A) HepG2 and (B) C2C12 cells.** Protein expression was assessed by Western blotting following treatment under high-glucose conditions. Data are mean RBD ± SEM (n = 3), normalised to β-actin. *p* < 0.05 vs HG.

Hyperglycemic conditions significantly increased p38 MAPK expression in HepG2 cells (1.72 ± 0.10-fold) compared to normoglycemic controls (*p* < 0.0001) (Fig. 6A). Treatment with *C. papaya* leaf extract reduced p38 MAPK expression at HGL500 (0.94 ± 0.06-fold; *p* = 0.0029) and HGL1000 (0.81 ± 0.05-fold; *p* = 0.0006). Root extract produced similar reductions at HGR500 (0.89 ± 0.04-fold; *p* = 0.0018) and HGR1000 (0.77 ± 0.06-fold; *p* = 0.0003). Metformin also significantly reduced p38 MAPK expression (0.83 ± 0.05-fold; *p* = 0.0011).

A comparable trend was observed for p38 MAPK expression in C2C12 cells (Fig. 6B). Hyperglycemia significantly increased p38 MAPK expression to 1.45 ± 0.05-fold relative to normoglycemic controls (*p* = 0.0265). However, treatment with metformin significantly reduced p38 expression to 0.80 ± 0.08-fold (*p* = 0.0063). Similarly, *C. papaya* leaf extract significantly suppressed p38 MAPK expression at HGL500 (0.52 ± 0.09-fold; *p* = 0.0026) and HGL1000 (0.45 ± 0.02-fold; *p* = 0.0030), while root extract induced significant reductions at HGR500 (0.61 ± 0.01-fold; *p* = 0.0038) and HGR1000 (0.82 ± 0.03-fold; *p* = 0.0018), relative to hyperglycemic controls.

### 3.4 IRS-1 and insulin signalling

**Fig. 7:**
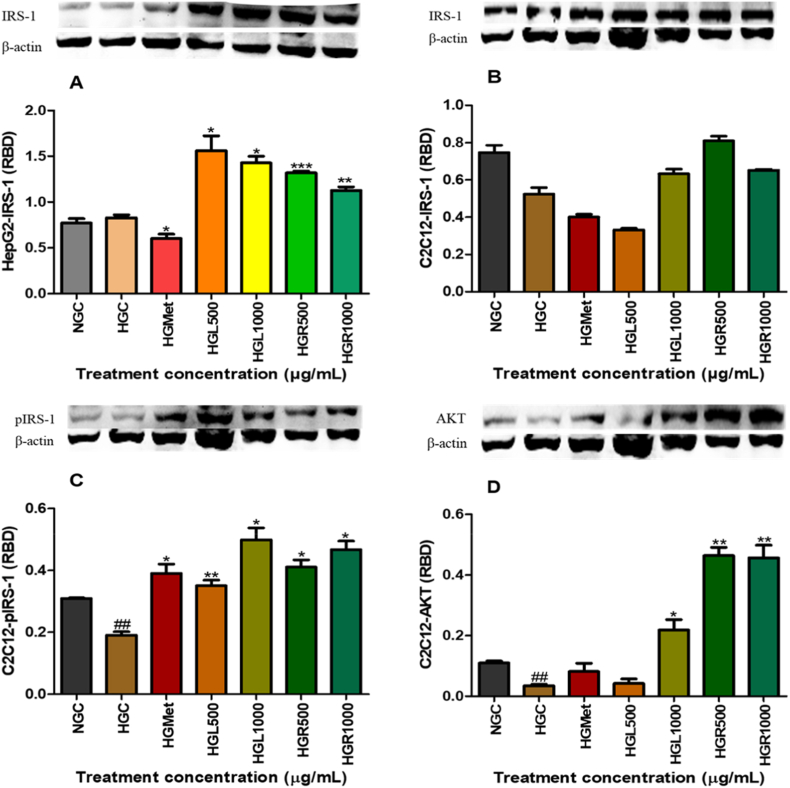
**Effects of *Carica papaya* extracts on insulin-signalling proteins in HepG2 and C2C12 cells.** (A) IRS-1 in HepG2 cells; (B) IRS-1 in C2C12 cells; (C) phosphorylated IRS-1 in C2C12 cells; (D) AKT in C2C12 cells. Proteins were analysed by Western blotting after treatment under hyperglycemic conditions. Values are mean RBD ± SEM (n = 3), normalised to β-actin. *p* < 0.05 vs HG.

Total IRS-1 protein expression was significantly reduced in hyperglycemic HepG2 cells (0.62 ± 0.05-fold) relative to normoglycemic controls (*p* < 0.001) (Fig. 7A). Treatment with *C. papaya* leaf extract significantly increased IRS-1 expression at HGL500 (1.09 ± 0.06-fold; *p* = 0.0012) and HGL1000 (1.18 ± 0.07-fold; *p* < 0.0001). Root extract also increased IRS-1 expression at HGR500 (1.02 ± 0.05-fold; *p* = 0.0036) and HGR1000 (1.21 ± 0.08-fold; *p* < 0.0001). Metformin-treated cells showed reduced IRS-1 expression compared to untreated controls (0.71 ± 0.06-fold; *p* = 0.0284).

Total IRS-1 expression in C2C12 cells was non-significantly reduced under hyperglycemic conditions (0.70 ± 0.05-fold) compared to normoglycemic controls. IRS-1 expression remained non-significantly altered following treatment with metformin (0.76 ± 0.03-fold; *p* > 0.05) and HGL500 (0.64 ± 0.02-fold; *p* > 0.05). In contrast, non-significant increases in IRS-1 expression were observed following treatment with HGL1000 (1.21 ± 0.05-fold), HGR500 (1.55 ± 0.05-fold), and HGR1000 (1.25 ± 0.01-fold) relative to HGC (Fig. 7B). Despite minimal changes in total IRS-1, phosphorylated IRS-1 levels were significantly increased following *C. papaya* treatment. The leaf extract increased pIRS-1 expression at HGL500 (1.85 ± 0.09-fold; *p* = 0.0045) and HGL1000 (2.62 ± 0.20-fold; *p* = 0.0163), while root extract induced significant increases at HGR500 (2.16 ± 0.12-fold; *p* = 0.0132) and HGR1000 (2.46 ± 0.14-fold; *p* = 0.0111), comparable to metformin (2.05 ± 0.16-fold; *p* = 0.0246) (Fig. 7C).

AKT protein expression was significantly reduced under hyperglycemic conditions (0.32 ± 0.05-fold; *p* < 0.01). Significant increases were observed following treatment with HGL1000 (2.01 ± 0.32-fold; *p* = 0.0340), HGR500 (4.27 ± 0.25-fold; *p* = 0.0040), and HGR1000 (4.18 ± 0.38-fold; *p* = 0.0097), whereas metformin and HGL500 did not significantly alter AKT expression (Fig. 7D).

### *3.5* *GLUT2* and *AKT* gene expression in HepG2 cells

**Fig. 8:**
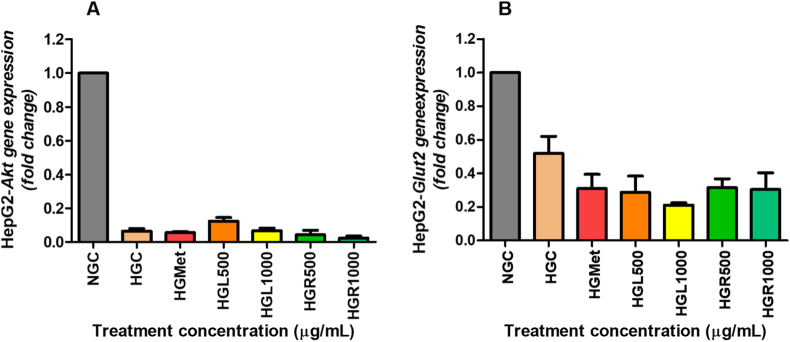
**Effect of *Carica papaya* extracts on (A) *AKT* and (B) *GLUT2* mRNA expression in HepG2 cells.** Gene expression was quantified by qPCR, normalised to GAPDH, and calculated using the ΔΔCt method. Data are expressed as fold change relative to HG and presented as mean ± SEM (n = 3). *p* < 0.05 vs HG.

In the untreated normal glucose control HepG2 cells, gene expression levels of GLUT2 and AKT were significantly reduced in the hyperglycemic treated cells (*p* < 0.05). Treatment with *C. papaya* leaf and root extracts led to a non-significant downregulation of *GLUT2* gene expression in HepG2 cells (*p* > 0.05), which was comparable to the effect of metformin when assessed against the hyperglycemic control (Fig. 8A). Additionally, while *C. papaya* leaf extracts resulted in a non-significant increase in AKT gene expression (*p* < 0.05). Treatment with *C. papaya* root extracts caused a non-significant decrease in *AKT gene* expression in HepG2 cells, mirroring the effect observed with metformin (*p* < 0.05) (Fig. 8B).

### 3.6 AMPKα activation protein expression in HepG2 and C2C12 cells

**Fig. 9:**
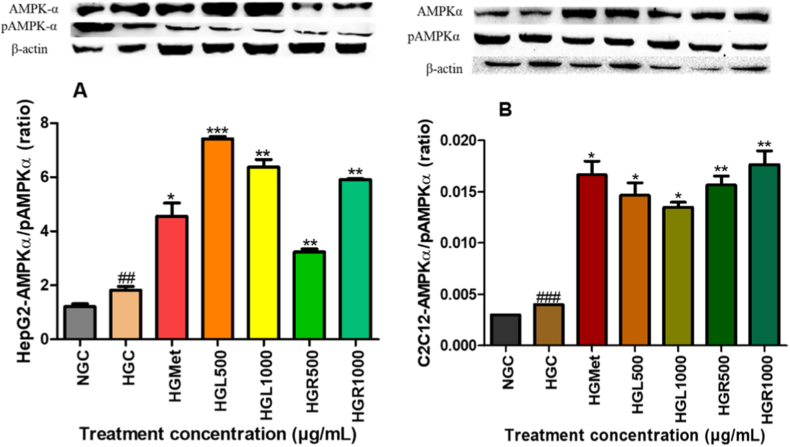
**Activation of AMPKα and phosphorylated AMPKα in (A) HepG2 and (B) C2C12 cells following *Carica papaya* treatment.** Protein levels were measured by Western blotting under hyperglycemic conditions and normalised to β-actin. Data are mean RBD ± SEM (n = 3). *p* < 0.05 vs HG.

A significant upregulation of AMPK expression was observed in HepG2 cells under hyperglycemic conditions, with HGC showing a 1.50-fold increase relative to normoglycemic controls (*p* = 0.0382) (Fig. 9A). Under hyperglycemic conditions, metformin treatment further increased AMPK expression to 2.49 ± 0.27-fold (*p* = 0.0330) relative to HGC. Similarly, *C. papaya* leaf extract induced a significant increases in AMPK expression at HGL500 (4.06 ± 0.05-fold; *p* < 0.0001) and HGL1000 (3.49 ± 0.16-fold; *p* = 0.0049), while root extract significantly increased AMPK expression at HGR500 (1.77 ± 0.06-fold; *p* = 0.0046) and HGR1000 (3.24 ± 0.02-fold; *p* = 0.0012) relative to hyperglycemic controls.

A comparable trend was observed in C2C12 cells (Fig. 9B), where hyperglycemia significantly increased AMPK expression by 1.33-fold compared to normoglycemic controls (*p* < 0.0001). Under hyperglycemic conditions, metformin further elevated AMPK expression to 5.56 ± 0.44-fold (*p* = 0.0109). Treatment with *C. papaya* leaf extract significantly increased AMPK expression at HGL500 (4.89 ± 0.40-fold; *p* = 0.0125) and HGL1000 (4.50 ± 0.17-fold; *p* = 0.0335), while root extract induced significant increases at HGR500 (5.22 ± 0.29-fold; *p* = 0.0057) and HGR1000 (5.89 ± 0.44-fold; *p* = 0.0094), relative to hyperglycemic controls.

### 3.7 Glycogen synthase expression on HepG2 and C2C12 cells

**Fig. 10:**
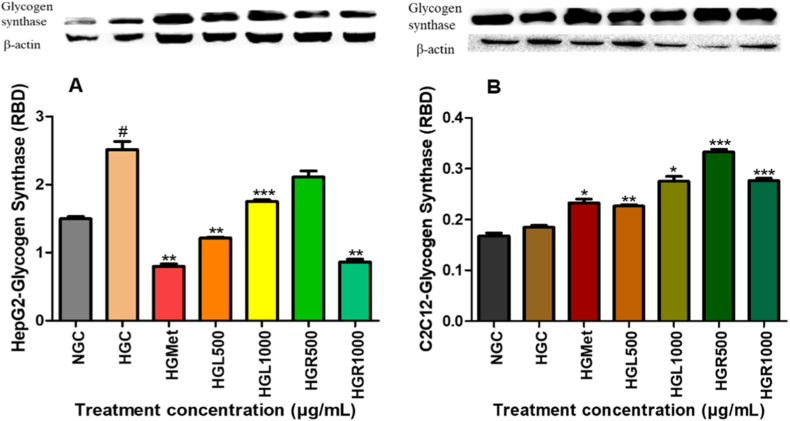
**Regulation of glycogen synthase (GS) expression by *Carica papaya* extracts in (A) HepG2 and (B) C2C12 cells.** GS protein expression was analysed by Western blotting after treatment under high-glucose conditions. Values are mean ± SEM (n = 3), normalised to β-actin. *p* < 0.05 vs HG.

In HepG2 cells, hyperglycemia significantly increased GS expression to 1.68 ± 0.08-fold relative to normoglycemic controls (*p* = 0.0145) (Fig. 10A). Treatment with metformin significantly reduced GS expression to 0.53 ± 0.03-fold (*p* = 0.0053). *C. papaya* leaf extract also reduced GS expression at HGL500 (0.81 ± 0.01-fold; *p* = 0.0085) and HGL1000 (0.70 ± 0.01-fold; *p* = 0.0248), while root extract significantly reduced GS expression at HGR1000 (0.57 ± 0.03-fold; *p* = 0.0058). The reduction observed at HGR500 (1.41 ± 0.06-fold) was not statistically significant.

In contrast, GS expression in C2C12 cells was not significantly altered by hyperglycemia alone. However, treatment with metformin increased GS expression to 1.26 ± 0.04-fold (*p* = 0.0327). *C. papaya* leaf extract significantly increased GS expression at HGL500 (1.23 ± 0.01-fold; *p* = 0.0030) and HGL1000 (1.49 ± 0.05-fold; *p* = 0.0131), while root extract induced robust increases at HGR500 (1.80 ± 0.03-fold; *p* = 0.0002) and HGR1000 (1.50 ± 0.03-fold; *p* = 0.0007) relative to hyperglycemic controls (Fig. 10B).

## 4 Discussion

Glucose homeostasis is primarily regulated by the liver and skeletal muscle, which together account for the majority of postprandial glucose uptake and storage [16]. Hepatic glucose uptake occurs largely in an insulin-independent manner via GLUT2, whereas skeletal muscle glucose disposal depends on insulin-stimulated GLUT4 translocation and downstream PI3K/AKT signalling ([5], Iksen et al., 2021). In T2DM, chronic hyperglycaemia disrupts these processes through impaired insulin signalling and sustained activation of stress-responsive pathways, particularly MAPKs, which promote insulin resistance [12,17]. In this study, *Carica papaya* leaf and root extracts were evaluated for their ability to modulate glucose-regulatory enzymes and intracellular signalling pathways under hyperglycaemic conditions in HepG2 hepatic cells and C2C12 skeletal muscle cells.

Inhibition of carbohydrate-digesting enzymes represents an established strategy for limiting excessive glucose availability and attenuating postprandial hyperglycaemia [31]. Consistent with this approach, *C. papaya* extracts significantly suppressed α-amylase activity in both HepG2 and C2C12 cells (Fig. 2), while exerting comparatively weaker effects on α-glucosidase activity (Fig. 3). This selective inhibition aligns with previous studies identifying *C. papaya* polyphenols, particularly quercetin, kaempferol, and caffeic acid derivatives, as potent α-amylase inhibitors [32,33]. Similar enzyme-inhibitory effects have been reported in diabetic animal models and *in vitro* systems, where *C. papaya* extracts reduced carbohydrate digestion and glucose availability [32,34]. Collectively, these findings support the notion that *C. papaya* may limit intracellular glucose overload under hyperglycaemic conditions by modulating extracellular carbohydrate metabolism.

MAPK signalling plays a central role in the development of insulin resistance by promoting inhibitory serine phosphorylation of insulin receptor substrate-1 (IRS-1), thereby impairing insulin receptor signalling [16,19,35]. In HepG2 cells, both *C. papaya* leaf and root extracts significantly reduced the expression of SAPK/JNK, Erk1/2, and p38 MAPK (Figs. 4A, 5A and 6B). Suppression of these kinases is mechanistically relevant, as JNK- and Erk-mediated IRS-1 phosphorylation has been strongly implicated in hepatic insulin resistance [[36], [37], [38], [39]]. Notably, MAPK inhibition in HepG2 cells was accompanied by restoration of IRS-1 protein levels (Fig. 7A), indicating relief from stress kinase–mediated repression. These observations are consistent with previous reports demonstrating that *C. papaya* attenuates MAPK-driven inflammatory signalling in hepatic and endothelial cell models, thereby improving insulin sensitivity under metabolic stress [30,40].

In C2C12 skeletal muscle cells, MAPK modulation exhibited a tissue-specific pattern. Although JNK expression was not uniformly suppressed (Fig. 4B), reductions in Erk1/2 and p38 MAPK were evident under most treatment conditions (Figs. 5B and 6B). Importantly, despite modest changes in total IRS-1 expression (Fig. 7B), *C. papaya* extracts significantly increased IRS-1 phosphorylation (Fig. 7C), indicating effective activation of downstream insulin signalling. These findings highlight the tissue-specific regulation of MAPK–IRS-1 crosstalk and emphasise the importance of independently evaluating hepatic and skeletal muscle glucose metabolism. Consistent with these observations, previous studies have demonstrated that *C. papaya* interferes with MAPK signalling by inhibiting Erk1/2, JNK, and p38 phosphorylation in inflammatory and stress-induced cellular models [40,41].

Activation of IRS-1 promotes downstream PI3K/AKT signalling, a central pathway for insulin-stimulated glucose uptake, particularly in skeletal muscle [42]. In HepG2 cells, *AKT* expression remained unchanged following treatment (Fig. 8A), suggesting that enhanced glucose handling occurred independently of AKT upregulation. This is consistent with the insulin-independent nature of hepatic glucose uptake and supports the involvement of alternative regulatory mechanisms [5]. This observation aligns with prior studies indicating that *C. papaya* may enhance hepatic glucose handling through AMPK-dependent and antioxidant mechanisms rather than direct AKT activation [29,30]. In contrast, *C. papaya* extracts significantly increased AKT protein expression in C2C12 cells (Fig. 7D), correlating with elevated IRS-1 phosphorylation (7C). These findings indicate that *C. papaya* extracts stimulate glucose uptake in skeletal muscle through activation of the classical PI3K/AKT pathway. These findings are in agreement with *in vivo* studies demonstrating restoration of AKT signalling and GLUT4 expression in skeletal muscle following *C. papaya* supplementation in diabetic and diet-induced insulin resistance models [28,43].

AMPK is a key metabolic sensor that enhances glucose uptake while suppressing anabolic storage pathways [20,44,45]. Both *C. papaya* extracts and metformin significantly increased AMPKα and phosphorylated AMPKα levels in HepG2 and C2C12 cells (Fig. 9A and B). In hepatic cells, elevated AMPK activity coincided with reduced glycogen synthase expression (Fig. 10A), suggesting that glucose taken up by hepatocytes was preferentially directed toward utilisation rather than storage. This metabolic shift is consistent with AMPK-driven suppression of glycogenesis under energy-stressed conditions [30]. In contrast, skeletal muscle cells exhibited increased GS expression following treatment with *C. papaya* extracts (Fig. 10B). This effect was accompanied by increased AKT expression (7D), supporting inhibition of glycogen synthase kinase-3β and subsequent activation of GS [6,7]. These findings indicate that *C. papaya* extracts promote glucose storage in skeletal muscle while favouring glucose utilisation in liver cells, reflecting physiologically appropriate, tissue-specific metabolic regulation.

**Fig. 11:**
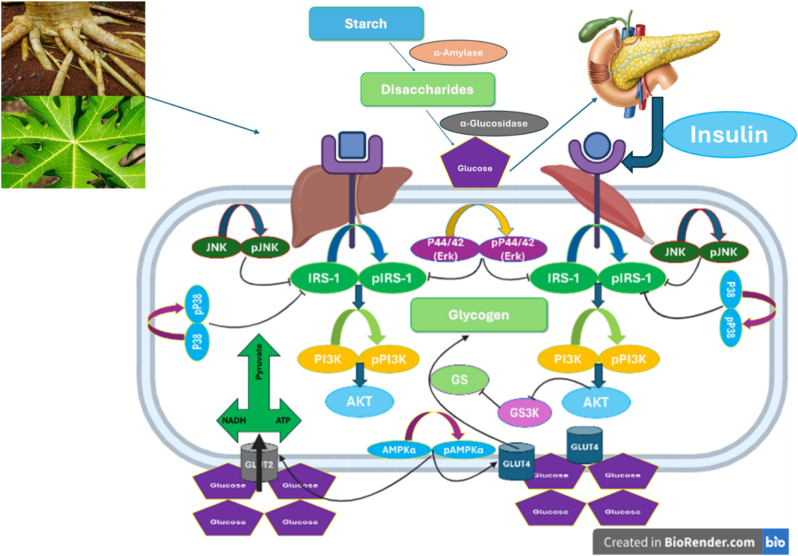
Schematic representation of the regulation of glucose uptake for usage and/or storage by *C. papaya* leaf and roots extracts in HepG2 liver and C2C12 muscle cells.

Taken together, the present findings demonstrate that *C. papaya* leaf and root extracts modulate glucose metabolism through coordinated inhibition of MAPK signalling and activation of IRS-1–dependent pathways. The differential responses observed in hepatic and skeletal muscle cells highlight the capacity of *C. papaya* to target multiple metabolic nodes relevant to insulin resistance and T2DM (see Fig. 11). When viewed alongside existing *in vitro, in vivo, and in silico* evidence, these results reinforce the therapeutic potential of *C. papaya*–derived bioactives as multi-target modulators of glucose homeostasis.

## 5 Conclusion

This study demonstrates that aqueous *Carica papaya* leaf and root extracts exert multi-level antidiabetic effects in hepatic (HepG2) and skeletal muscle (C2C12) cells under hyperglycemic conditions. The extracts significantly suppressed extracellular α-amylase activity in both cell lines and reduced α-glucosidase activity in muscle cells, indicating a capacity to limit glucose availability during hyperglycaemia. At the signalling level, *C. papaya* consistently attenuated MAPK activation while enhancing AMPK- and PI3K/AKT-associated pathways, supporting improved insulin sensitivity through cell-specific mechanisms. While glucose utilisation was inferred from molecular and metabolic markers rather than directly measured, the coordinated regulation of insulin-responsive pathways provides strong mechanistic support for enhanced glucose handling. Future studies incorporating functional glucose-uptake assays, glucose transporter dynamics, and differentiated muscle models will further refine these findings. Overall, this work provides compelling evidence that *C. papaya* extracts modulate key enzymatic and signalling targets relevant to insulin resistance, supporting their potential as complementary agents for glycaemic regulation.

## Informed consent statement

Not applicable.

## Institutional review board statement

Ethical approval was obtained from the Biomedical Research Ethics Administration under Ethics Number: BREC/00005903/2023; approved: December 31, 2023.

## Declaration of AI use

During the preparation of this manuscript, the authors used an artificial intelligence–assisted language model (ChatGPT, OpenAI) to support language editing, clarity, and organization of the text. The AI tool was used solely to improve readability and coherence. All scientific content, data interpretation, and conclusions were critically reviewed and validated by the authors, who take full responsibility for the integrity and originality of the work.

## Funding

This research was funded by the 10.13039/501100024216College of Health Sciences, University of Kwa-Zulu Natal, Durban, South Africa, and the 10.13039/501100001321South African National Research Foundation (NRF) (PMDS22070532834).

## CRediT authorship contribution statement

**Mthokozisi Bongani Nxumalo:** Conceptualization, Data curation, Formal analysis, Funding acquisition, Investigation, Methodology, Project administration, Software, Validation, Visualization, Writing – original draft, Writing – review & editing. **Rene Bernadette Khan:** Data curation, Funding acquisition, Project administration, Resources, Supervision, Validation, Writing – review & editing. **Nosipho Ntanzi:** Conceptualization, Data curation, Methodology, Validation, Writing – review & editing. **Fave Yohanna Tata:** Data curation, Methodology, Validation, Writing – review & editing. **Hezekiel Mathambo Kumalo:** Conceptualization, Data curation, Funding acquisition, Project administration, Resources, Supervision, Validation, Visualization, Writing – review & editing.

## Declaration of competing interest

The authors declare that they have no known competing financial interests or personal relationships that could have appeared to influence the work reported in this paper.

## Data Availability

The data generated was used to support the findings of this study and is included in the article.
